# Reaction–diffusion theory explains hypoxia and heterogeneous growth within microbial biofilms associated with chronic infections

**DOI:** 10.1038/npjbiofilms.2016.12

**Published:** 2016-06-22

**Authors:** Philip S Stewart, Tianyu Zhang, Ruifang Xu, Betsey Pitts, Marshall C Walters, Frank Roe, Judith Kikhney, Annette Moter

**Affiliations:** 1 Center for Biofilm Engineering, Montana State University, Bozeman, MT, USA; 2 Chemical and Biological Engineering, Montana State University, Bozeman, MT, USA; 3 Mathematical Sciences, Montana State University, Bozeman, MT, USA; 4 Biofilmcenter, German Heart Institute Berlin, Berlin, Germany

## Abstract

Reaction–diffusion models were applied to gain insight into the aspects of biofilm infection and persistence by comparing mathematical simulations with the experimental data from varied bacterial biofilms. These comparisons, including three *in vitro* systems and two clinical investigations of specimens examined *ex vivo*, underscored the central importance of concentration gradients of metabolic substrates and the resulting physiological heterogeneity of the microorganisms. Relatively simple one-dimensional and two-dimensional (2D) models captured the: (1) experimentally determined distribution of specific growth rates measured in *Pseudomonas aeruginosa* cells within sputum from cystic fibrosis patients; (2) pattern of relative growth rate within aggregates of streptococcal biofilm harboured in an endocarditis vegetation; (3) incomplete penetration of oxygen into a *Pseudomonas aeruginosa* biofilm under conditions of exposure to ambient air and also pure oxygen; (4) localisation of anabolic activity around the periphery of *P. aeruginosa* cell clusters formed in a flow cell and attribution of this pattern to iron limitation; (5) very low specific growth rates, as small as 0.025 h^−1^, in the interior of cell clusters within a *Klebsiella pneumoniae* biofilm in a complex 2D domain of variable cell density.

## Introduction

The list of chronic infections stemming from biofilms continues to grow as does awareness of the economic and human toll incurred by these debilitating infections. Reaction–diffusion theory has been successfully applied for decades to understand microscale chemical gradients, ecological niches and substrate fluxes in wastewater treatment and environmental biofilms. There are far fewer examples of the adaptation of this theory to systems of medical relevance. Here the general utility of this class of models, which derive from first principles of concerted Fickian diffusion and metabolic reaction, is illustrated with case studies involving limitation for oxygen, glucose and iron. Recurrent themes of hypoxia, physiological heterogeneity in the microbial population and antibiotic tolerance resulting from non-growing cells emerge. Altogether, these examples show that reaction–diffusion theory can be applied to shed light on the chemical and physiological heterogeneity that likely contributes to the pathogenesis and persistence of biofilm infections.

Mathematical modelling of biofilms has contributed to the understanding of the functioning of these aggregated microbial communities since the seminal article of Enrique Lamotta in 1976.^1^ Hundreds of biofilm modelling papers have been published subsequently, of which we cite here only a few of the landmark examples.^2–9^ Almost all of these studies were motivated by applications in water and wastewater treatment. They address the operation of systems such as trickling filtres, anaerobic sludge digesters and drinking water distribution pipes.

As the awareness of the role of biofilm formation in numerous persistent infections expands,^10–13^ an opportunity exists to enhance the understanding of the formation, activity and ecology of infectious biofilms by adapting the same types of mathematical models that have been so successful in civil engineering application to the systems of medical and dental interest. This opportunity remains relatively unexplored and underdeveloped. There are a few pioneering examples of biofilm modelling applied to medical systems.^14–24^ These models have contributed important insights into such topics as the mechanism of dental caries,^15,23^ the penetration of antibiotics into biofilms,^14,17^ the induction of quorum sensing in a biofilm^19^ and probiotic control of a pathogenic biofilm.^22^

The purpose of the work reported in this article is to demonstrate the general applicability of reaction–diffusion theory to biofilm problems of medical relevance. We focus in particular on the capacity of this theoretical approach to predict gradients in the concentration of metabolic substrates and, as a consequence, patterns of microbial growth that are spatially heterogeneous.

## Results

### Case 1—prediction of the spatial distribution of oxygen within a biofilm and how it depends on the concentration of oxygen applied to the surface of the biofilm

**Figure 1:**
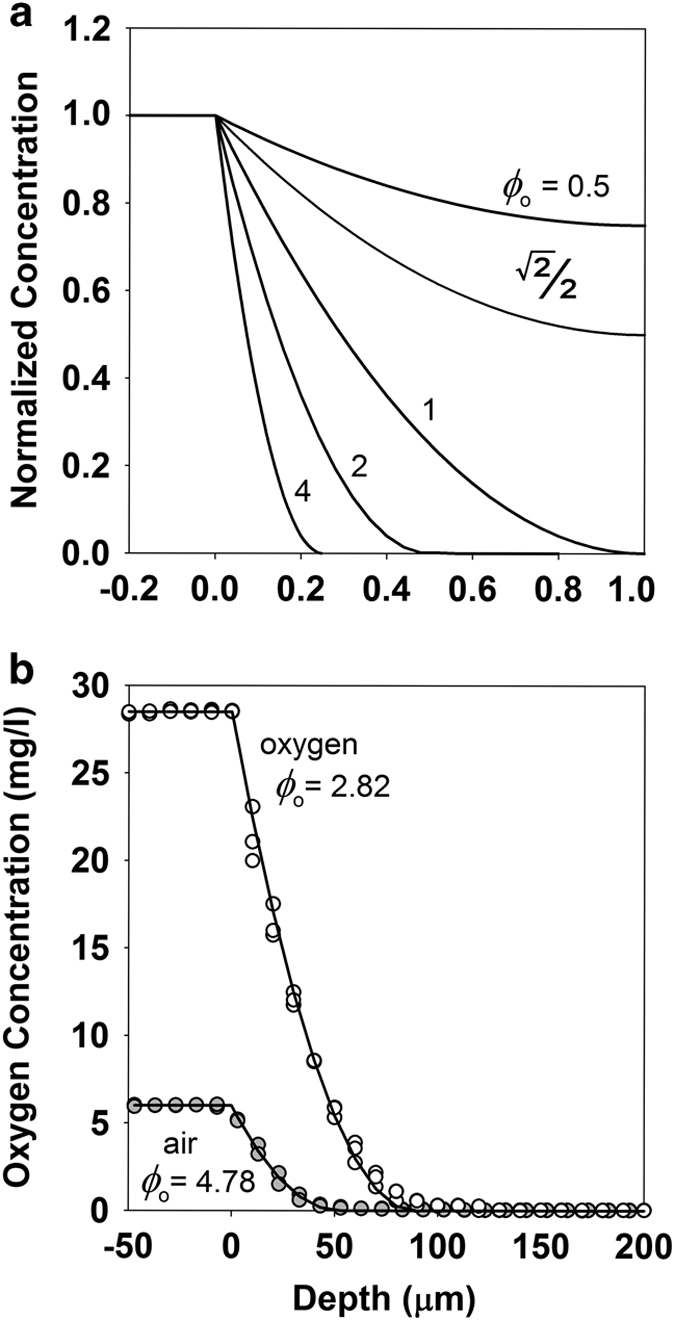
Experimental and theoretical oxygen concentration profiles in a *P. aeruginosa* colony biofilm. (**a**) Theoretical concentration profiles (solid curves) for a metabolic substrate experiencing zero-order reaction kinetics for varying values of the Thiele modulus, *ϕ*_o_. (**b**) Experimental oxygen concentration profiles in a single *P. aeruginosa* colony biofilm exposed to either air (grey circles) or oxygen-enriched gas (open circles). The solid lines are theoretical curves fit to the two data sets.

Because oxygen is sparingly soluble and is rapidly respired by aerobic microorganisms, oxygen concentration gradients are a common feature of biofilm systems. The oxygen profile measured in a simple *in vitro P. aeruginosa* biofilm describes a curve whose shape is fit reasonably well by the parabolic solution to the zero-order reaction–diffusion problem (Figure 1a). In this example, a value of the Thiele modulus of *ϕ*_o_=2.82 provides an acceptable description of the data (Figure 1b). The experimental measurements plotted in Figure 1 are from a single-colony biofilm that was first profiled in air then flooded with pure oxygen gas and profiled again in this oxygen-enriched environment. The measurements were performed in the same exact planar location in the biofilm. Thus, the fixed physical (*L*_f_, *ρ*, *D*_e_) and intrinsic biological parameters (*μ*_o_, *Y*_xs_) that constitute *ϕ*_o_ were identical. The only difference between the air and oxygen conditions was owing to the change in the concentration of oxygen at the boundary (*C*_o_). We predict therefore that the ratio of *ϕ*_o_ values between these two cases should be numerically equal to the square root of the ratio of the bulk oxygen concentrations (see Equation (4)), which is (28.5/6)^1/2^ or 2.18. The actual ratio of the two fitted values of *ϕ*_o_ was 4.78/2.82 or 1.70. The Thiele modulus values determined in air and oxygen were statistically significantly different (*P*<10^−4^).

### Case 2—calculation of the probability distribution of specific growth rates in a biofilm

**Figure 2:**
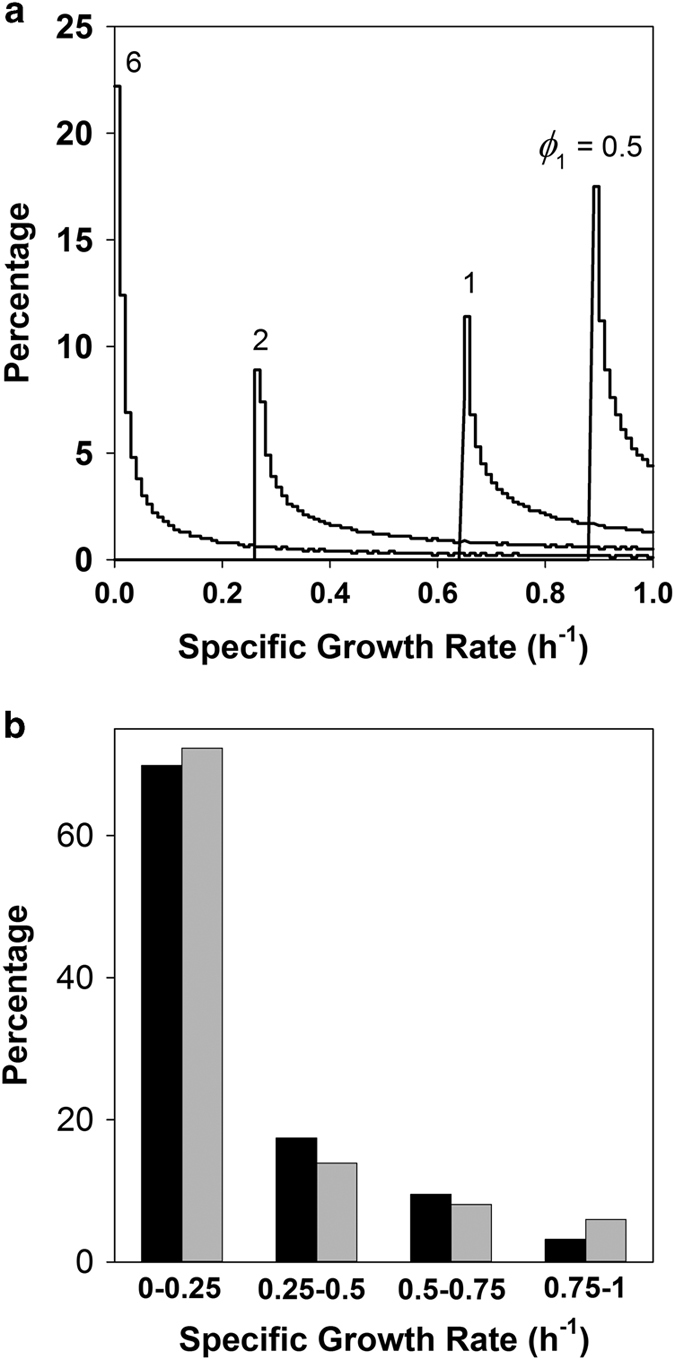
Predicted distribution of bacterial growth rates in a biofilm. (**a**) Growth rate distributions for a flat slab biofilm governed by first-order reaction kinetics for varying values of the Thiele modulus, *ϕ*_1_. (**b**) Comparison of measured growth rate distribution of *P. aeruginosa* in the explanted lungs of three CF patients^25^ (black bars) with the distribution predicted for *ϕ*_1_=5 (grey bars).

Given that biofilms harbour gradients in the concentration of the growth-limiting substrate, there will also be corresponding changes in the local cellular specific growth rate. For the case of first-order reaction kinetics this leads to variation in the growth rate in space that is captured in the distributions plotted in Figure 2a. When diffusion is only slightly limiting (*ϕ*_1_=0.5) growth rates are rapid and close to the growth rate that would be expected with the bulk fluid concentration of substrate (~1 h^−1^). As diffusion limitation increases (*ϕ*_1_ progressively larger) the distribution becomes broader and includes more lower values of growth rate.

Kragh *et al.* ^25^ recently reported single-cell measurements of specific growth rate of *P. aeruginosa* in the sputum of three patients with cystic fibrosis. We pooled these 63 measurements and created a quadripartite distribution. The theoretical distribution for a value of *ϕ*_1_=5 matches this experimental result well (Figure 2b).

### Case 3—visualisation and quantification of a growth rate gradient within a biofilm and identification of the growth-limiting substrate as iron

One way to visualise gradients in anabolic activity within a biofilm is by using a bacterial strain containing an inducible fluorescent protein. A time-lapse microscopy video sequence of such an experiment can be viewed in the Supplementary Movie S1. In this experiment, a *P. aeruginosa* biofilm containing an isopropylthio-β-D-galactoside-inducible green fluorescent protein (GFP) was grown for 5 days in the absence of the inducer. The biofilm was dark at this point. The inducing agent was then added to the medium, in uninterrupted continuous flow. Over the next few hours, green colour developed in the cell cluster corresponding to the local expression of GFP. More GFP was expressed at the periphery of the cluster than at the cluster center. This gradient reflects the relative growth rate of the bacteria in different regions of the cluster.

**Figure 3:**
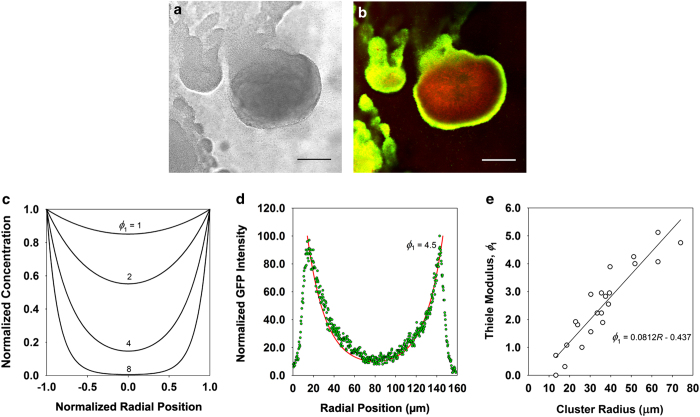
Relative growth rate patterns in round clusters of *P. aeruginosa* biofilm. (**a**) Transmission image of a cell cluster. (**b**) Pattern of GFP induction (green) and biomass counterstain (red) in the same cell cluster. **a** and **b** were imaged through the glass attachment surface in the plane of the substratum scale bars are 50 microns. (**c**), Theoretical substrate concentration profiles in a hemispherical cluster subject to first-order reaction kinetics for varying values of the Thiele modulus, *ϕ*_1_. (**d**) Theoretical curve (*ϕ*_1_=4.5) fitted to an experimental GFP fluorescence intensity obtained by image analysis from an experiment like that shown in **b**. (**e**) Thiele modulus determined by fit to experimental data plotted versus cluster radius (symbols). The line is a least squares regression whose slope has the value (*k*_1_/*D*_e_)^1/2^; see Equation (17).

The process of quantifying the pattern of growth within an experiment like that described above is illustrated in Figure 3. A biofilm cluster imaged in transmission mode was ~126 μm in diameter (Figure 3a). After the induction of GFP and counterstaining with a red dye, a pattern of GFP expression similar to that described above was observed: brighter green near the edges of the cluster and dimmer green towards the center (Figure 3b). The red stain reveals the distribution of biomass independent of metabolic activity. Calculated concentration profiles (Equation (18)) of a reacting substrate in a hemispherical cluster are given in Figure 3c for several values of the Thiele modulus, *ϕ*_1_. Note that as the growth rate in this case is directly proportional to the local substrate concentration, the spatial patterns calculated in Figure 3c are expected to apply to the microbial-specific growth rate as well as the substrate concentration itself. Image analysis of the green fluorescence intensity within a biofilm cluster can be compared with the theoretical patterns like those in Figure 3c to extract a quantitative estimate of the Thiele modulus. An example of such a fit is shown in Figure 3d.

When this image analysis process was applied to multiple biofilm clusters (*n*=22) from several experiments, a range of values of *ϕ*_1_ were determined (Figure 3e). The estimated Thiele modulus increased with the measured radius of the cell cluster (Figure 3e). This linear dependence is expected from the definition of the Thiele modulus. The slope of the line fitted to the data in Figure 3e provides access to a quantitative estimate of the value of the first-order reaction rate coefficient, *k*_1_.

This discussion has so far not addressed the identity of the growth-limiting substrate. Indeed, the observation of an activity gradient within a biofilm does not by itself provide any clue as to the nature of the limitation. The likely identity of the growth-limiting substrate can be accessed by comparing values of *k*_1_ derived from experiment (i.e., Figure 3e) and calculated *a priori* from independent estimates of the constituent parameters. These comparisons are summarised in Table 1. All of the theoretical estimates made use of the same value of the growth rate of the bacteria under bulk fluid conditions (*μ*_o_). The growth rate at 23 °C of this *P. aeruginosa* strain in the minimal medium used was measured in batch culture to be 0.16 h^−1^. The theoretical estimates also used the same constant value of the cell density in the biofilm (*ρ*) of 10^4^ mg l^−1^. Yield coefficients (*Y*_xs_) were estimated based on the typical composition of biomass and reference to measured values.^26^ The bulk fluid concentration of the limiting substrate (*C*_o_) was determined by the medium composition for carbon and nitrogen and by the solubility of oxygen in water at the typical barometric pressure in Bozeman, Montana. Iron concentration was estimated as the solubility limit of iron(III) phosphate.^27^ Diffusion coefficients in the biofilm were estimated as described elsewhere.^28,29^ Comparison of the experimental values of *k*_1_ with the calculated theoretical values reveals a discrepancy for carbon, nitrogen and oxygen of two orders of magnitude or more. Only for iron is there a reasonable correspondence. Iron is thus the likely growth-limiting substrate in this case.

### Case 4A—calculation of spatial variation in growth rate within a heterogeneous biofilm *in vitro*

**Figure 4:**
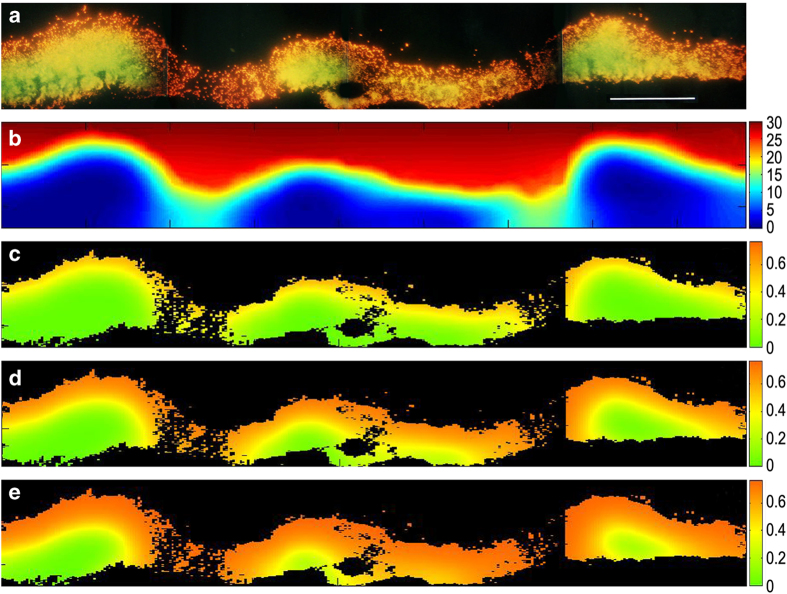
Simulated spatial patterns of specific growth rate in a 2D heterogeneous biofilm structure compared with an *in vitro* result. (**a**) Acridine orange stained frozen section showing regions of relatively rapid (red/orange) and slow (green/yellow) growth (reprinted with permission from ref. 30). (**b**) Calculated glucose concentration (mg l^−1^) for a bulk fluid glucose concentration of 30 mg l^−1^. (**c**–**e**) The predicted specific growth rate (h^−1^) for bulk fluid glucose concentrations of 10 mg l^−1^, (**c**); 25 mg l^−1^, (**d**); and 40 mg l^−1^, (**e**). Bar=100 μm.

Wentland *et al.* ^30^ reported an experimental visualisation of the relative growth rate within a *K. pneumoniae* biofilm grown *in vitro* in a continuous flow reactor. This result is reproduced in Figure 4a. The biofilm varied in thickness and local cell density. There was a band of active growth (indicated by orange or red colour) that tracked the biofilm-bulk fluid interface. Where the biofilm was thinner and less dense, the entire biofilm thickness was growing rapidly as indicated by the hotter colours. Where the biofilm was locally thicker, the interior of the clusters suggested lower growth rates as indicated by the cooler colours of yellow and green.

We simulated the distribution of the growth-limiting substrate, glucose, within a two-dimensional representation of the biofilm using parameter values summarised in Supplementary Table S2. An example of the predicted glucose concentration is shown in Figure 4b. This simulation shows that glucose concentrations are diminished within the three larger biofilm clusters. As the bulk fluid glucose concentration was varied (this being the one parameter for which an independent experimental estimate was not available), the predicted pattern of growth rates within the biofilm changed. At a relatively low bulk concentration of 10 mg l^−1^, growth rates in the biofilm were also relatively slow as indicated by a predominance of green and yellow hues (Figure 4c). At the highest bulk glucose concentration simulated, 40 mg l^−1^, growth rates were higher and much of the biofilm was predicted to be growing rapidly (Figure 4e). As the reactor influent concentration of glucose was 40 mg l^−1^, this constitutes an upper bound to the actual concentration experienced by the biofilm. The simulation at a bulk fluid glucose concentration of 25 mg l^−1^ (Figure 4d) resembles the experimental pattern (Figure 4a). Predicted growth rates manifested as a clear spatial gradient within the biofilm ranging from 0.71 h^−1^ (84% of the maximum growth rate) to just 0.025 h^−1^ (3% of maximum).

### Case 4B—calculation of spatial variation in growth rate within a heterogeneous biofilm *ex vivo*

**Figure 5:**
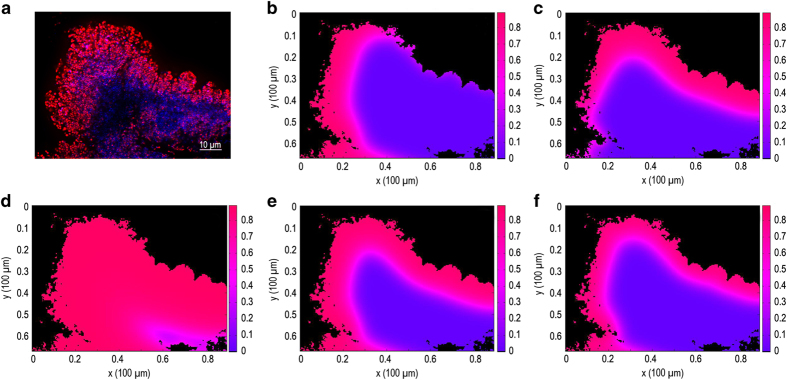
Simulated spatial patterns of specific growth rate in a 2D heterogeneous biofilm structure compared with an *ex vivo* result. (**a**) FISH-probed human clinical endocarditis specimen showing cells with relatively rapid (red), intermediate (magenta), or slow or no activity (blue) as indicated by relative ribosome content. (**b**–**f**) The predicted specific growth rate (h^−1^) for bulk fluid glucose concentrations of 100 mg l^−1^, (**b**,**c**,**e**); 500 mg l^−1^, (**d**); and 50 mg l^−1^, (**f**). Glucose was provided at the left boundary (**b**), top boundary (**c**), or on both the left and top boundaries (**d**–**f**).

We analyse here the pattern of activity found in a fluorescence *in situ* hybridisation (FISH)-probed infectious biofilm recovered from the heart valve vegetation of an endocarditis patient (Figure 5a). The microorganism was determined to be *Streptococcus equinus* using 16S rRNA (ribosomal RNA)-gene sequencing. The relative ribosome content as revealed by the fluorescence signal intensity associated with the amount of hybridised FISH probe (red colour) indicated localised activity associated with the left and top boundaries of the biofilm. Spatial patterns of bacterial growth within this structure were simulated using parameter values summarised in Supplementary Table S3. Simulations that only modelled provision of glucose from the left or top boundaries alone (Figures 5b,c, respectively) were unable to produce qualitatively accurate representations of the observed activity pattern. Rather, it was necessary to model the provision of the growth-limiting nutrient, glucose, from both the left and top boundaries (Figures 5d–f).

A starting point for setting the glucose concentration at the boundaries was the glucose concentration in human plasma, ~900 mg l^−1^ (ref. 31). When high concentrations of glucose were assumed along the left and top boundaries of the aggregate, rapid microbial growth was predicted throughout the biofilm (Figure 5d). Only when the boundary-condition glucose concentration was reduced to ~100 mg l^−1^ or less (Figures 5e and f), did the predicted patterns conform to the observed pattern. This result shows that this biofilm aggregate was likely exposed to lower concentrations of nutrient than are available in plasma, probably as a result of external mass transfer limitations.

## Discussion

Reaction–diffusion theory can be applied to shed light on the chemical and physiological heterogeneity that likely contributes to the pathogenesis and persistence of biofilm infections. Here we have analysed elementary problems that illustrate the propensity for hypoxia in the vicinity of a biofilm, the potential for limitation by diverse substrates from oxygen to glucose to iron, and the reality of distributed growth states in the microbial population that range from rapidly growing to dormant.

The comparison reported in Figure 1b can be regarded as a test of the potential of hyperbaric oxygen therapy to improve the penetration of oxygen into a biofilm. The penetration depth of oxygen is predicted to increase as the square root of the applied oxygen concentration. For example, quadrupling the oxygen tension only doubles the penetration depth of oxygen. This result highlights a possible limitation to the efficacy of delivering oxygen into an infectious biofilm with hyperbaric oxygen therapy.^32^ This limitation may not be widely recognised in the medical field even though the concentration dependence of substrate penetration has been known for decades in the context of biofilm wastewater treatment process.^33^

Hypoxia is a recurrent theme in biofilm infections.^34–36^ It impacts healing, the oxidative burst of neutrophils and bacterial antibiotic tolerance.^37^ Reaction–diffusion models are an appropriate approach in analysing hypoxia, though they will need to go beyond the simple models presented here to incorporate oxygen transport in the vasculature and oxygen consumption by host tissue and leukocytes.

Quantitative results derived from a reaction–diffusion analysis allowed us to diagnose an example of iron limitation (Figure 3). Iron has not previously been identified as a limiting substrate for biofilm growth to our knowledge. Iron limitation is quite plausible *in vivo*, however, where most iron is sequestered.^38^ The very simple minimal medium that was used in this experiment, unlike many laboratory media, contained no added trace elements. The only iron present probably entered as a contaminant of the constituent salts. Because of the relatively high concentration of phosphate, iron would have precipitated as an iron phosphate. Precipitated iron that remains in suspension will be accessible to planktonic cells, but in the biofilm only the dissolved iron would be able to access the interior of a cell cluster by diffusion.

Our calculations show that microscale biological heterogeneity in specific growth rate can be predicted from first principles of reaction and diffusion (Figures 2, 4 and 5). This heterogeneity is evident in the variation in growth rate between individual cells in a population (Figure 2) and in spatial patterns across multicellular aggregates of bacteria (Figures 4 and 5). In these three examples—cystic fibrosis sputum, an *in vitro* Enterobacterial biofilm and a bacterially colonised endocarditis vegetation—the biofilm not only harbours growing bacterial cells, but also cells that are growing slowly or not at all. This result may aid in understanding antibiotic tolerance arising from biofilms that harbour a significant proportion of non-growing bacteria.^39^

## Materials and methods

### Theory

The solutions for Cases 1, 2, and 3 are well-known in the technical literature.^40,41^ We recap them here and pose them in terms of microbiological and biofilm parameters. Nomenclature and units are provided in Supplementary Table S1.

### Case 1

Consider a flat slab biofilm of uniform thickness in which the growth-limiting substrate is consumed subject to zero-order kinetics. The spatial variable, *z*, is oriented such that *z*=0 at the biofilm-bulk fluid interface and *z*=*L*_f_ corresponds to the substratum or attachment surface. The dimensionless spatial variable is *ξ*=*z*/*L*_f_. A differential balance on this substrate, when non-dimensionalised, yields:

(1)d2udξ2=2ϕo2

where the two terms correspond to reaction (right) and diffusion (left) and *u* denotes dimensionless substrate concentration. Boundary conditions impose a fixed concentration of substrate at the surface of the biofilm and a no-flux condition at the point in the biofilm at which substrate is depleted:

(2)u=1atξ=0

(3)dudξ=0atξ=aLf

The dimensionless parameter *ϕ*, termed the Thiele modulus, is a measure of the relative rates of reaction and diffusion. Because the particular form of *ϕ* depends on the kinetic form of the reaction, we use a subscript to denote the kinetic form to avoid confusion. For the zero-order kinetic problem, the Thiele modulus, interpreted in terms of microbial growth and substrate consumption in a biofilm, is

(4)ϕo=(µmaxρLf22CoYxsDe)1/2

The solution (the concentration profile within the biofilm) is given by

(5)u={ϕo2ξ2−2ϕoξ+1,0≤ξ≤1ϕo0,1ϕo≤ξ≤1

and the penetration depth of the substrate is

(6)a=Lfϕo,

(7)a∝Co12.

Note that this solution applies when the biofilm is thick enough that the substrate is completely depleted at some point (*ϕ*_o_>1). A different solution applies for biofilms that are fully penetrated by the substrate:

(8)u=ϕo2ξ2−2ϕo2ξ+1.

### Case 2

Consider a flat slab biofilm of uniform thickness in which the growth-limiting substrate is consumed subject to first-order kinetics. The microbial growth rate is proportional to the substrate concentration: *μ*=*μ*_o_ *u*. Note in this case that the spatial variable, *z*, runs in the opposite direction from the analysis in Case 1. Here *z*=0 corresponds to the bottom of the biofilm or substratum and *z*=*L*_f_ corresponds to the biofilm-bulk fluid interface. The non-dimensionalised spatial variable is *ξ*=*z*/*L*_f_. The differential balance on substrate, in non-dimensional form, is

(9)d2udξ2=ϕ12u

with boundary conditions

(10)u=1atξ=1

(11)dudξ=0atξ=0.

These boundary conditions impose a fixed concentration at the surface of the biofilm and a no-flux condition at the substratum.

The Thiele modulus is given by

(12)ϕ1=(µoρLf2CoYxsDe)1/2.

And the concentration profile is given by

(13)u=cosh(ϕ1ξ)coshϕ1.

Growth rates calculated from this concentration profile were binned to construct growth rate distributions.

### Case 3

Consider a hemispherical biofilm cluster in which the growth-limiting substrate is consumed subject to first-order kinetics. The spatial variable, *r*, runs from *r*=0 at the center of the cluster to *r*=*R* at the surface of the cluster of biofilm-bulk fluid interface and is made dimensionless with *ξ*=*r*/*R*. The differential balance on substrate, in non-dimensional form is

(14)1ξ2ddξ(ξ2dudξ)=ϕ12u

with boundary conditions,

(15)u=1atξ=1

(16)dudξ=0atξ=0

which impose a fixed concentration at the surface of the biofilm and a finite concentration at the cluster center, respectively.

The Thiele modulus in this case is given by

(17)ϕ1=(k1R2De)1/2=(µoρR2CoYxsDe)1/2.

And the solution is

(18)u=Rrsinh(ϕ1ξ)sinh(ϕ1).

### Case 4

In the preceding three cases, the cell density and effective diffusion coefficient have been assumed to be constant and uniform in space and simple (one-dimensional) geometries have been assumed. Now we relax these constraints to analyse heterogeneous biofilm structures like those that occur in actual biofilms formed in nature, *in vitro,* or *in vivo*.^42,43^ In a two-dimensional field of width *X* and height *Y*, the unsteady differential balance on substrate gives

(19)∂C∂t=∂∂x(D∂C∂x)+∂∂y(D∂C∂y)−µmaxρCYxs(KM+C)

where Monod growth kinetics have been assumed. Note that the parameters *D*_e_ and *ρ* are allowed to vary with *x* and *y*.^44,45^ *ρ*=*ρ*(*x*,*y*) is the local cell density, which is obtained based on the experimental grey-scale image as follows. At each pixel, we assign a cell density proportional to the light intensity of the image such that the average cell density is *ρ*. If the total number of pixels is *N*, and the *i*th pixel intensity is *I*_*i*_, then the cell density at the *i*th pixel is given by

(20)ρi=ρ(xi,yi)=IiN∑i=1NIiρ.

We note that *ρ* depends on space only and in the bulk fluid *ρ*_i_=0 since *I*_i_=0.

The diffusion coefficient is given by

(21)D={De,inbiofilmDb,inbulkfluid

where *D*_b_ is an apparent diffusion coefficient that has been artificially elevated so as to account for convective mixing that occurs in the moving fluid adjacent to the biofilm. *D*_e_ is the effective diffusive permeability of glucose inside the biofilms which depends on space only. *D*_e_=*D*_e_ (*x*,*y*) is calculated based on the approach derived by Hinson and Kocher^46^ as outlined in (ref. 47), and expressed by the equations below. In this formulation, *D*_eo_ is the effective diffusive permeability of the extracellular matrix, *D*_c_ is the diffusive permeability of the cell phase and *D*_p_ is the diffusive permeability of the pure extracellular polymer. *ϵ*_aq_, *ϵ*_c_ and *ϵ*_p_ denote the volume fractions of water, cells and extracellular polymers, respectively. Here at the *i*th pixel, the cell volume fraction is *ϵ*_c_=*ρ*/*ρ*_in_, where *ρ*_in_ is the cell intrinsic density.

(22)DeDaq=(DeDeo)(DeoDaq)

(23)DeDeo=2DaqDc+DaqDeo−2ϵc(DaqDc−DaqDeo)2DaqDc+DaqDeo+ϵc(DaqDc−DaqDeo)

(24)DeoDaq=ϵaq(ϵpDaqDp+ϵaq)−1

(25)b=ϵpϵc+ϵp

(26)ϵaq+ϵc+ϵp=1

The boundary conditions are

(29)∂C∂x=0for0<y<Yatx=0andx=X

(28)∂C∂y=0for0<x<Xaty=0

(29)C=Cofor0<x<Xaty=Y

which stipulate no flux conditions along the bottom and sides and a constant concentration along the top boundary.

The unsteady reaction–diffusion equation was solved on a rectangular spatial domain by the finite element method with discretisation in space and backward Euler difference in time. The computation was terminated when the relative change in *C* between consecutive time steps was <10^−3^, corresponding to a steady state. The microbial growth rate was calculated as

(30)µ=µmaxCKM+C

where *C* is the local steady state concentration of the growth-limiting substrate.

### Oxygen concentration profiles in colony biofilms

These methods pertain to Case 1. The Case 1 assumption of zero-order reaction kinetics is an appropriate approximation when the Monod half-saturation coefficient for the substrate is smaller than the concentration at the surface of the biofilm. This is the case for consumption of oxygen by *Pseudomonas aeruginosa* when the oxygen concentration at the biofilm interface is in equilibrium with air.^48,49^ *Pseudomonas aeruginosa* FRD1 colony biofilms were grown for 48 h and profiled with oxygen microelectrodes as previously detailed.^50^ To perform the experiment in an atmosphere enriched in oxygen, two holes were cut in the plastic lid of a Petri dish that was placed over the tryptic soy agar plate on which the colony biofilm rested. One hole was at the perimeter of the dish and allowed a silicone tube to be inserted, through which pure oxygen was delivered in a continuous stream. The second hole was centered in the lid and directly above the colony biofilm. Oxygen gas exited from this hole, which also enabled microelectrode access to the biofilm. A single-colony biofilm was first profiled in air, then flooded with oxygen gas as described above for 15 min and profiled again in this oxygen-enriched environment. The measurements were performed in the same spot in the biofilm (in duplicate in air and triplicate in oxygen). The oxygen concentration in equilibrium with air was measured to be 6.0 mg l^−1^ and with the oxygen-enriched environment was 28.5 mg l^−1^.

The parabolic solution given by Equation (5) was fitted to experimentally measured concentration profiles by least squares regression to estimate *ϕ*_o_ with the value of *C*_o_ fixed at the experimental value measured in the gas phase. A *t*-test of statistically significant difference between the values of *ϕ*_o_ determined in air and oxygen was implemented for the non-linear least squares problem.^51^

### Spatial pattern of GFP induction in flow-cell biofilms

These methods pertain to Case 3. *Pseudomonas aeruginosa* biofilms were grown in 1 mm square glass capillaries as described previously.^52^ Strain PAO1 containing plasmid pAB1 (ref. 53) was used. This strain carries a stable GFP under the control of the *trc* promoter, which is inducible with isopropylthio-β-D-galactoside. The minimal growth medium contained: 5 mmol l^−1^ glycerol, 0.9 mmol l^−1^ sodium glutamate, 145 mmol l^−1^ NaCl, 3.4 mmol l^−1^ K_2_HPO_4_, 1.5 mmol l^−1^ NaH_2_PO_4_·2H_2_O and 0.2 mmol l^−1^ MgSO_4_·7H_2_O. Biofilms were grown under continuous flow at ambient temperature (23 °C) for 5 days prior to induction with 1 mmol l^−1^ isopropylthio-β-D-galactoside. Some biofilms were counterstained with 50 mg l^−1^ rhodamine B for 30 min after the induction step to reveal biomass that did not express GFP. Images were collected with a Leica TCS-NT confocal scanning laser microscope using a 488-nm laser for excitation of GFP and a 561-nm laser for excitation of rhodamine B. Quantitative image analysis was performed using the Linescan function of MetaMorph software (Molecular Devices, Sunnyvale, CA, USA).

The data analysis from these experiments began by extracting an estimate of the first-order Thiele modulus, *ϕ*_1_, by dividing the GFP intensity at the center of a cell cluster by the intensity at the cluster edge. This ratio corresponds to the value of Equation (18) in the limit as *r* or *ξ* goes to zero, which is 1/sinh*ϕ*_1_. Thiele modulus values thus extracted were plotted versus cluster radius. From Equation (17), the slope of this line is (*k*_1_/*D*_e_)^1/2^. Then *k*_1,ex_ was found by multiplying the slope, determined by least squares regression, times the independently estimated effective diffusion coefficient.

### FISH of endocarditis specimens

These methods pertain to Case 4B. Heart valve tissue was fixed immediately after excision in the operating room, embedded in methacrylate and sectioned as described before.^54^ The heart valve of the endocarditis patient was investigated by FISH and 16S rRNA-gene-PCR and sequencing as part of the diagnostic routine workup of endocarditis. This use was exempt from human subjects approval and the related image included in the present study was fully anonymised. FISH was carried out using the *Streptococcus*-specific probe STREP1-Cy3 (false coloured red in Figure 5)^55^ combined with unspecific nucleic acid stain DAPI (4*'*,6- diamidino-2-phenylindole dihydrochloride) to visualise all bacterial cells (false coloured blue in Figure 5).^56^ Microscopic examination of the tissue sections was performed using an epifluorescence microscope (AxioPlan II, Zeiss, Jena, Germany) equipped with a 100 W high-pressure mercury lamp and narrow band filtre sets. Image acquisition was performed with an AxioCam MRm black and white camera and the AxioVision 4.6 software. Consecutive sections were submitted to DNA extraction followed by broad-range 16S rRNA-gene-PCR and sequencing as described.^57^

## Figures and Tables

**Table 1:** Comparison of experimental (*k*_1,ex_) and theoretical (*k*_1,th_) values of the first-order reaction rate coefficient for different limiting substrates in a *P. aeruginosa* biofilm

*Limiting substrate*	*D*_*e*_ *(cm* ^*2*^ *s* ^*−1*^)	*k*_*1,ex*_ *(s* ^*−1*^)	*(g*_*x*_ */g*_*s*_ *) Y*_*xs*_	*C*_*o*_ *(mg l* ^*−1*^)	*k*_*1,th*_ *(s* ^*−1*^ *)*
C	2.23×10^−6^	1.47	0.25	180	0.010
N	1.80×10^−6^	1.19	7.1	12.6	0.005
O_2_	11.4×10^−6^	7.52	0.85	6.0	0.087
Fe	3.41×10^−6^	2.25	500	0.00043	2.07

Substrates are glycerol (C), glutamate (N), molecular oxygen (O_2_) and Fe^2+^ (Fe). The medium concentrations, *C*_o_, are as the element. Diffusion coefficients in the biofilm were estimated as described elsewhere.^52,53^ Yield coefficients (*Y*_xs_) were estimated based on the typical composition of biomass and reference to measured values.^50^
