# Supplementary material for: Reaction–diffusion theory explains hypoxia and heterogeneous growth within microbial biofilms associated with chronic infections
Source: NPJ Biofilms Microbiomes. 2016 Jun 22;2:16012–. doi: 10.1038/npjbiofilms.2016.12 (PMC5515263; doi:10.1038/npjbiofilms.2016.12)
Supplement: Supplementary Information [file npjbiofilms201612-s1.doc]

**Table S1**. Nomenclature. L, length; M, mass; T, time.

| Symbol | Parameter | Units |
| --- | --- | --- |
| *a* | substrate penetration depth into biofilm | L |
| *b* | parameter used in calculating *D*_e_ | none |
| *C* | substrate concentration | M_s_ L^-3^ |
| *C*_o_ | substrate concentration at biofilm surface | M_s_ L^-3^ |
| *D* | diffusion coefficient | L T^-2^ |
| *D*_aq_ | diffusion coefficient in water | L T^-2^ |
| *D*_b_ | diffusion coefficient in bulk fluid | L T^-2^ |
| *D*_c_ | diffusion coefficient in cells | L T^-2^ |
| *D*_e_ | effective diffusion coefficient in biofilm | L T^-2^ |
| *D*_p_ | diffusion coefficient in polymer | L T^-2^ |
| *k*_1_ | first-order rate constant | T^-1^ |
| *K*_M_ | Monod half-saturation coefficient | M_s_ L^-3^ |
| *L*_f_ | biofilm thickness | L |
| *r* | radial spatial coordinate | L |
| *R* | biofilm cluster radius | L |
| *t* | time | T |
| *u* | dimensionless concentration (*C*/*C*_o_) | none |
| *Y*_xs_ | yield coefficient of biomass on substrate | M_x_ M_s_^-1^ |
| *z* | Cartesian spatial coordinate | L |
| *ϵ*_aq_ | volume fraction of water phase | none |
| *ϵ*_c_ | volume fraction of cells | none |
| *ϵ*_p_ | volume fraction of polymers | none |
| *μ* | specific growth rate | T^-1^ |
| *μ*_max_ | maximum specific growth rate | T^-1^ |
| *μ*_o_ | specific growth rate at bulk fluid conditions | T^-1^ |
| *ξ* | dimensionless spatial coordinate | none |
| *ρ* | biofilm cell density (mass per biofilm volume) | M_x_ L^-3^ |
| *ρ_in_* | Intrinsic cell density (mass per cell volume) | M_x_ L^-3^ |
| *ϕ*_0_ | Thiele modulus, zero-order kinetics | none |
| *ϕ*_1_ | Thiele modulus, first-order kinetics | none |

**Table S2**. Parameter values for simulation Case 4A.

| Symbol | Parameter | Value | Source |
| --- | --- | --- | --- |
| *b* | parameter used in calculating *D*_e_ | 0.15 | 1 |
| *D*_aq_ | glucose diffusion coefficient in water | 6.7 x 10^-6^ cm^2^ s^-1^ | 2 |
| *D*_e_ | apparent glucose diffusion coefficient in bulk fluid accounting for advection | 6.7 x 10^-5^cm^2^ s^-1^ | assumed |
| *D*_p_ /*D*_aq_ | parameter used in calculating *D*_e_ | 0.02 | 1 |
| *D*_c_ /*D*_aq_ | parameter used in calculating *D*_e_ | 0.1 | 1 |
| *h*_o_ | characteristic length scale | 100 μm | assumed |
| *K*_M_ | Monod coefficient | 3.5 mg l^-1^ | 2 |
| *X* | domain size in x | 881 μm | image size |
| *Y* | domain size in y | 125 μm | image size |
| *C*_o_ | bulk fluid glucose concentration | 1 ~ 40 mg l^-1^ | 2 |
| *t*_o_ | characteristic time scale | 3600 s | assumed |
| *ρ*_avg_ | average cell density | 2 x 10^4^ mg l^-1^ | 2 |
| *ρ*_in_ | intrinsic cell density | 4 x 10^5^ mg l^-1^ | 2 |
| *Y*_xs_ | yield coefficient of biomass on glucose | 0.45 g_x_ g_s_^-1^ | 3 |
| *μ*_max_ | maximum specific growth rate | 0.84 h^-1^ | 2 |

1. Stewart, P. S. A review of experimental measurements of effective diffusive permeabilities and effective diffusion coefficients in biofilms. *Biotechnol. Bioeng.* **59,** 261-272 (1998).
2. Stewart, P. S., Camper, A. K., Handran, S. D., Huang, C.-T. & Warnecke, M. Spatial distribution and coexistence of *Klebsiella pneumoniae* and *Pseudomonas aeruginosa* in biofilms. Microb. Ecol. **22,** 2-10 (1997).
3. Neijssel, O. M. & Tempest, D. W. 1975. The regulation of carbohydrate metabolism in *Klebsiella aerogenes* NCTC 418 organisms, growing in chemostat culture. *Arch. Microbiol.* **106,** 251-258 (1975).

**Table S3**. Parameter values for simulation Case 4B.

| Symbol | Parameter | Value | Source |
| --- | --- | --- | --- |
| *b* | parameter used in calculating *D*_e_ | 0.15 | 1 |
| *D*_aq_ | glucose diffusion coefficient in water | 9.0 x 10^-6^ cm^2^ s^-1^ | 2 |
| *D*_e_ | apparent glucose diffusion coefficient in bulk fluid accounting for advection | 9.0 x 10^-5^cm^2^ s^-1^ | assumed |
| *D*_p_ /*D*_aq_ | parameter used in calculating *D*_e_ | 0.02 | 1 |
| *D*_c_ /*D*_aq_ | parameter used in calculating *D*_e_ | 0.1 | 1 |
| *h*_o_ | characteristic length scale | 100 μm | assumed |
| *K*_M_ | Monod coefficient | 5.0 mg l^-1^ | 3 |
| *X* | domain size in x | 89.2 μm | image size |
| *Y* | domain size in y | 66.8 μm | image size |
| *C*_o_ | bulk fluid glucose concentration | 900 mg l^-1^ | 4 |
| *t*_o_ | characteristic time scale | 3600 s | assumed |
| *ρ*_avg_ | average cell density | 2 x 10^5^ mg l^-1^ | 2 |
| *ρ*_in_ | intrinsic cell density | 4 x 10^5^ mg l^-1^ | 2 |
| *Y*_xs_ | yield coefficient of biomass on glucose | 0.45 g_x_ g_s_^-1^ | 2 |
| *μ*_max_ | maximum specific growth rate | 0.90 h^-1^ | 5 |

1. Stewart, P. S. A review of experimental measurements of effective diffusive permeabilities and effective diffusion coefficients in biofilms. *Biotechnol. Bioeng.* **59,** 261-272 (1998).
2. Stewart, P. S., Camper, A. K., Handran, S. D., Huang, C.-T. & Warnecke, M. Spatial distribution and coexistence of *Klebsiella pneumoniae* and *Pseudomonas aeruginosa* in biofilms. Microb. Ecol. **22,** 2-10 (1997).
3. Neijssel, O. M. & Tempest, D. W. 1975. The regulation of carbohydrate metabolism in *Klebsiella aerogenes* NCTC 418 organisms, growing in chemostat culture. *Arch. Microbiol.* **106,** 251-258 (1975).
4. Blanch, H. W. & Clark, D. S. Biochemical Engineering. Marcel Dekker, New York, NY (1997).
5. Lehninger, A. L. Biochemistry. Worth Publishers, New York, NY (1975).
6. Carvalho, S. M., Kuipers, O. P., Neves, A. R. Environmental and nutritional factors that affect growth and metabolism of the pneumococcal serotype 2 strain D39 and its nonencapsulated derivative strain R6. *PLoS One* **8,** e58492 (2013).
